# Planning and evaluating mental health services in low- and middle-income countries using theory of change

**DOI:** 10.1192/bjp.bp.114.153841

**Published:** 2016-01

**Authors:** Erica Breuer, Mary J. De Silva, Rahul Shidaye, Inge Petersen, Juliet Nakku, Mark J. D. Jordans, Abebaw Fekadu, Crick Lund

**Affiliations:** **Erica Breuer**, MPH, Alan J Flisher Centre for Public Mental Health, Department of Psychiatry and Mental Health, University of Cape Town, Cape Town, South Africa; **Mary J. De Silva**, BA, MSc, PhD, Centre for Global Mental Health, London School of Hygiene and Tropical Medicine, London, UK; **Rahul Shidaye**, MBBS, MD, Public Health Foundation of India, Bhopal, Madhya Pradesh, India and Maastricht University/CAPHRI School for Public Health and Primary Care, Maastricht, The Netherlands; **Inge Petersen**, BSc, BSc(Hons), MSc, PhD, School of Applied Human Sciences, University of KwaZulu-Natal, Howard College Campus, Durban, South Africa; **Juliet Nakku**, MBChB, MMed, Butabika National Mental Hospital, Kampala, Uganda; **Mark J. D. Jordans**, MSc, PhD, HealthNet TPO, Amsterdam, The Netherlands and King's College London, Institute of Psychiatry, Psychology and Neuroscience, London, UK; **Abebaw Fekadu**, MD, PhD, MRCPsych, King's College London, Institute of Psychiatry, Psychology and Neuroscience, London, UK and Addis Department of Psychiatry, College of Health Sciences, Addis Ababa University, Addis Ababa, Ethiopia; **Crick Lund**, BA, BSocSci, MA, MSocSci, PhD, Alan J Flisher Centre for Public Mental Health, Department of Psychiatry and Mental Health, University of Cape Town, Cape Town, South Africa, and Centre for Global Mental Health, Institute of Psychiatry, Psychology and Neuroscience, King's College London, UK

## Abstract

**Background**

There is little practical guidance on how contextually relevant mental healthcare plans (MHCPs) can be developed in low-resource settings.

**Aims**

To describe how theory of change (ToC) was used to plan the development and evaluation of MHCPs as part of the PRogramme for Improving Mental health carE (PRIME).

**Method**

ToC development occurred in three stages: (a) development of a cross-country ToC by 15 PRIME consortium members; (b) development of country-specific ToCs in 13 workshops with a median of 15 (interquartile range 13–22) stakeholders per workshop; and (c) review and refinement of the cross-country ToC by 18 PRIME consortium members.

**Results**

One cross-country and five district ToCs were developed that outlined the steps required to improve outcomes for people with mental disorders in PRIME districts.

**Conclusions**

ToC is a valuable participatory method that can be used to develop MHCPs and plan their evaluation.

Despite growing recognition that mental health services should be integrated into primary care substantial constraints to integration exist in low- and middle-income countries (LMIC).^1^ These include competing public health priorities,^2^ low investment in mental health services,^3^ a paucity of specialist human resources^4^ and resistance to decentralisation.^2^ There is also little practical guidance on how planning for integrated mental health services can be achieved. The World Health Organization (WHO) mental health policy and service guidance package,^5^ for example, provides overall guidance on the steps to follow in the development of mental health policies and plans but does not provide detail on how this can be done in practice to develop contextually relevant MHCPs. As part of the PRogramme for Improving Mental health carE (PRIME) we used theory of change (ToC) as an approach to developing integrated mental healthcare plans (MHCPs) for specific districts in Ethiopia, India, Nepal, South Africa and Uganda.^6,7^ Using PRIME as a case study, this article describes how ToC was used as a planning tool for the development and evaluation of the PRIME MHCPs and provides a framework that can be adapted for use in the development of MHCPs in low-resource settings.

## Method

### The ToC approach

ToC is a theory-driven approach to programme design and evaluation that starts by making explicit a theory of how a programme will achieve its impact by describing the hypothesised steps along the causal pathway and uses this theory to guide the evaluation of the programme.^8^ It has been used to design and evaluate complex programmes,^9–13^ including systems of mental healthcare for children.^14^ However, based on preliminary results of a systematic review conducted by the authors, there are no reported examples in the literature of how it can be used for the development of mental health services in LMIC.

The defining feature of a ToC compared with the logframe or logic models^13^ is that ToC organises the short-, medium- and long-term outcomes necessary to achieved the impact outcome onto a causal pathway, or ToC map.^15^ This impact is the long-term vision of the programme and will often occur long after the programme is completed.^16^ The activities or interventions required to move from one outcome to the next are mapped onto the causal pathway. The evidence base or rationale for each link in the causal pathway is made explicit, usually based on literature reviews or the tacit knowledge of implementers. Assumptions about the conditions under which the ToC will work are articulated as part of the ToC. In addition, indicators are developed for each outcome along the causal pathway in order to measure progress. The ToC approach is purposefully method-neutral and does not prescribe the types of evaluation designs that are used to collect the indicators for the ToC.^8^ Ideally, a ToC should be developed during the planning stages^8^ of a programme using various methods: reviews of programme documentation, interviews with stakeholders and/or stakeholder workshops.^12^

### The ToC development process in PRIME

We used a ToC approach as one of the methods to develop the PRIME MHCPs. This occurred in three overlapping stages (Table 1). The first stage involved the development of an initial cross-country ToC at a workshop in India in November 2011, involving 15 key PRIME partners including psychiatrists, psychologists, epidemiologists, programme managers and at least two people who were experienced in mental health service delivery in each of the PRIME countries.

**Table 1:** Stages of theory of change (ToC) development process in the PRogramme for Improving Mental health carE (PRIME)

	Stage	Participants, *n*
*1. Initial development of PRIME cross-country ToC*		
November–December 2011	i. PRIME cross-country ToC workshop with key PRIME partners	15

*2. Development of district-specific ToCs*		
a. Sodo, Ethiopia		
February 2012	i. Pre-ToC workshop with PRIME Ethiopia team	10
February 2012	ii. ToC workshop with community- and district-level representatives	17
February 2012	iii. Final ToC workshop with national-level planners	13
b. Sehore, India		
December 2011	i. Development of trial ToC by PRIME India group	4
January 2012	ii. ToC workshop with district and health facility representatives	20
April 2012	iii. ToC workshop with national-level planners	17
c. Chitwan, Nepal		
February 2012	i. ToC workshop with district and health facility representatives	14
March 2012	ii. ToC workshop with national-level planners	10
March 2012	iii. ToC workshop with district and health facility representatives	11
April 2012	iv. ToC workshop at national-level planners	8
d. Dr Kenneth Kuanda, South Africa		
March 2012	i. ToC workshop with health-facility-, district-, provincial- and national- level representatives	38
March 2012	ii. ToC workshop with community-level representatives	26
August 2012	iii. ToC workshop with community-, health facility-, district-, provincial- and national-level representatives	37
e. Kamuli, Uganda		
February 2012	i. ToC workshop with district and health facility representatives	22
July 2012	ii. ToC workshop with district and health facility representatives	22

*3. Refinement of PRIME cross-country ToC*		
December 2011	i. Review of ToC by other PRIME members	17
October 2012–April 2013	ii. Review of country ToCs and revision of cross-country ToC	Led by 2 consortium members with written feedback from consortium
October 2012–March 2013	iii. ToC and mental healthcare plan indicator mapping	Led by 3 consortium members 3 with written input from >1 researcher from each country

The purpose of the ToC workshop was to introduce PRIME partners to the ToC approach and to develop and refine the PRIME cross-country ToC. This included mapping out the hypothesised causal pathways that comprised key outcomes and interventions necessary to achieve effective coverage of evidence-based mental health services and the ultimate impact of PRIME. The identified impact was: improved health, social and economic outcomes for people living with priority mental disorders and their families/carers in the district.

The ToC was informed by the previous work and principles of the PRIME consortium including the following.

- The guiding principles of PRIME:
  - a focus on health systems strengthening;
  - working in partnership with Ministries of Health;
  - prioritising key mental disorders;
  - developing robust frameworks for the design and evaluation of complex interventions; and
  - ensuring equity.^6^
- A draft framework for the PRIME MHCPs developed by the PRIME consortium at the outset of the project that outlined the three levels of the health system at which integration of mental health into primary care should occur: healthcare organisation, health facility and community-based care.^6^
- Work undertaken during the development of the WHO Mental Health Gap Action Programme (mhGAP)^17,18^ and the *PLoS Medicine* series on packages on mental, neurological and substance use disorders in LMIC^19^ to identify cost-effective interventions.
- The programmatic and research experience of the PRIME partners.
- A situational analysis of the PRIME districts.^20^

During the workshop, the participants also identified a range of assumptions required to successfully implement the MHCP. These assumptions identified the contextual conditions that needed to be in place for the MHCP to function or which might limit or facilitate the implementation of the MHCPs. These assumptions were used to identify cross-country research questions that were developed into cross-country interview guides for semi-structured interviews and focus-group discussions with stakeholders and adapted for use in PRIME countries.

The second stage of the ToC process was the development of specific ToCs for each PRIME implementation districts: Sodo, Ethiopia; Sehore, India; Chitwan, Nepal;^21^ Dr Kenneth Kuanda, South Africa; and Kamuli, Uganda. Details regarding the characteristics of the district sites have been provided elsewhere in this supplement.^22–26^ These were developed primarily using ToC workshops with stakeholders in each district, informed by Andersen's guidelines on conducting ToC workshops.^15^ The structure, content and stakeholders in the workshops have been described in detail elsewhere.^27^ In brief, between two and four ToC workshops were held in each PRIME country with a median of 15 (interquartile range 13–22) stakeholders per workshop including policy makers, district-level health planners and management, mental health specialists, researchers, service providers and, in some countries, patients (Table 1). The stakeholder composition of the workshops was determined by the PRIME country teams in order to include key decision makers and take into account the hierarchical nature of the local context. The ToC maps for each district were subsequently refined in different ways in each country by the PRIME country research teams using results of other formative work, ongoing internal meetings and meetings with stakeholders.

Following the development of the district-specific ToCs, the content of the MHCPs were developed for each district.^22–26^ The ToC was used as a framework to identify interventions that would be feasible in the setting, the human and other resources that could be used to provide these interventions, the contextual barriers and facilitating factors for implementation and the indicators to measure success. The ToC workshops provided rich discussions on many of the above issues and allowed aspects of the plan to be refined and agreed upon by key stakeholders.^27^ However, given the length of the ToC workshops and the number of stakeholders, not all details of the MHCP could be discussed and were finalised using other formative work including results from qualitative formative research among stakeholders and piloting.^7^ For example, in South Africa, interviews with stakeholders were used to inform the cultural appropriateness and acceptability of interventions such as using HIV counsellors to provide depression counselling in a group format. Similarly in Nepal, the formative work helped to identify the types of stakeholders who could assist with community detection. In Ethiopia, piloting the training of healthcare workers helped to determine the amount of practical time included in the training.

The third stage of the ToC process involved the refinement of the cross-country ToC. This started with a review of the cross-country ToC by key members of the PRIME consortium who were not present at the initial development workshop. The feedback was provided at a PRIME meeting in December 2011 where 17 members of the PRIME consortium were present, through individual discussions and email correspondence. In addition, we conducted a review of the district-specific ToC maps to ensure that the PRIME cross-country ToC covered all the major pathways and assumptions outlined in the district ToCs. We also developed indicators for each outcome of the ToC to measure whether the outcomes are achieved. The interventions that are required for one outcome to lead to the next were mapped onto the PRIME cross-country ToC. Then we began consolidating the input, process, output and outcome indicators developed for the interventions for all five of the implementation districts. This was done by looking across all five sets of MHCP indicators and choosing key indicators that were common across the packages and could be implemented in all countries. These indicators were combined into a master list that was reviewed by the members of the consortium. Each of the indicators was operationalised and study designs were chosen that would measure these indicators. The methods used to evaluate the MHCPs are described in detail elsewhere in this supplement.^28^

## Results

**Fig. 1:**
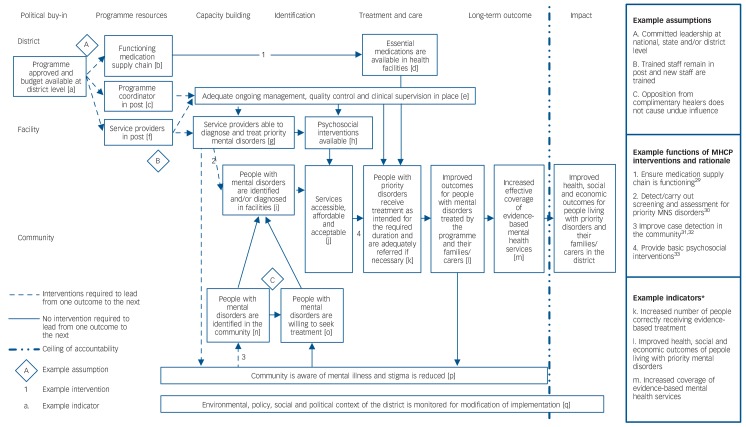
The PRogramme for Improving Mental health carE (PRIME) cross-country summary theory of change (ToC). *Example indicators for the summary TOC are outlined in Table 3 and Table 4. MHCP, mental healthcare plan; MNS, mental, neurological and substance use.

The PRIME ToC process resulted in various outputs including six ToCs: one cross-country ToC and five district ToCs (Table 2). The main components of the cross-country ToC are outlined below, namely the outcomes pathway, the key interventions, the major assumptions and the indicators, with a summary ToC map illustrated in Fig. 1. A more detailed version of the cross-country ToC can be found in online Fig. DS1. Following this, we describe the key differences between the cross-country ToC and the district-specific ToCs that can be found in online Figs DS2–6.

**Table 2:** Outputs from the theory of change (ToC) development process

Stage of development and outputs
1. Initial development of PRogramme for Improving Mental health carE (PRIME) cross-country ToC Preliminary PRIME cross-country ToC Cross-country interview guides for the individual in-depth interviews and focus-group discussions with stakeholders

2. Development of district-specific ToCs District-specific ToCs for Sodo, Ethiopia (online Fig. DS2); Sehore, India (online Fig. DS3); Chitwan, Nepal (online Fig. DS4); Dr Kenneth Kuanda, South Africa (online Fig. DS5); Kamuli, Uganda (online Fig. DS6) District-specific mental healthcare plans for Sodo, Ethiopia;^22^ Sehore, India;^23^ Chitwan, Nepal;^24^ Kamuli, Uganda;^25^ Dr Kenneth Kuanda, South Africa^26^

3. Refinement of PRIME cross-country ToC PRIME cross-country ToC (online Fig. DS1) Outline of PRIME evaluation design (see De Silva *et al*^28^ in this supplement for more details)

### The PRIME cross-country ToC matrix

The underlying structure of the PRIME ToC is a matrix, with level of the health system on the vertical axis describing where the MHCPs are implemented (community, health facility and healthcare organisation), and the temporal dimension on the horizontal axis illustrating the sequence in which the MHCPs are implemented. The temporal dimension should be read from left to right and specifies the types of outcomes along the hypothesised causal pathway required to reach the desired impact (Fig. 1). Specifically: getting political buy-in; mobilisation of programme resources; capacity building; identification of people with mental disorders; treatment and care; and long-term outcome and impact. The short-, medium- and long-term outcomes required to reach the impact are specified on the ToC map with the indicators for each outcome and how they will be measured described in Tables 3 and 4. The point at which the programme is no longer responsible for the outcome is delineated by a ‘ceiling of accountability’.

**Table 3:** Theory of change (ToC) indicators at health organisation level

ToC outcome	Indicator(s)	Study design
a. Programme approved and budget available at district level	Mental health integrated into the district health plan % increase in financial resources allocated to mental health released on time and available to spend	Case study: district profile

b. Functioning medication supply chain	Number of stockouts in past 30 days for essential psychotropic medications outlined in the mental healthcare plan	Case study: facility profile

c. Programme coordinator in post	Mental health programme coordinator in post prior to mental healthcare plan implementation	Case study: district profile

d. Essential medications are available in health facilities	Medications are available at all clinics 95% of time (disaggregated by clinic and type of medication)	Case study: facility profile

e. Adequate ongoing management, quality control and clinical supervision in place	All staff receive quality supervision on a regular basis as defined by the mental healthcare plan and guidelines	Case study: training and supervision evaluation Case study: process evaluation

**Table 4:** Theory of change (ToC) indicators at facility and community levels

ToC outcome	Indicator(s)	Study design
f. Service providers in post	Adequate numbers of human resources as per the mental healthcare plan are available at primary and community levels	Case study: facility profile

g. Service providers able to diagnose and treat priority mental disorders	Change in knowledge and attitudes pre- and post-training	Case study: training and supervision evaluation

h. Psychosocial interventions available	Staff trained in psychosocial interventions are available at the facility	Case study: facility profile

i. People with mental disorders are identified and/or diagnosed in facilities	Increased number and proportion of people correctly identified/ diagnosed with depression and alcohol use and treated with evidence-based interventions Increase in % mental health case-load as a proportion of total primary healthcare headcount	Facility detection survey Case study: facility profile

j. Services accessible, affordable and acceptable	Patients' perception of accessibility and acceptability of services	Qualitative cohort Cohort

k. People with priority disorders receive treatment as intended for the required duration and adequately referred if necessary	Increased number of people correctly receiving evidence-based treatment Number of patients who received psychosocial interventions at community level and facility level for the required duration	Facility detection survey Cohort

l. Improved outcomes for people with mental disorders treated by the programme and their families/carers	Improved health, social and economic outcomes of people living with priority mental disorders	Cohort

m. Increased effective coverage of evidence- based mental health services	Increased coverage of evidence-based mental health services	Community survey Cohort

n. People with mental disorders are identified in the community	Increased number of cases detected and managed by community health workers	Case study: community profile

o. People with mental disorders are willing to seek treatment	Increase in help-seeking and earlier presentation at clinic	Facility detection survey

p. Community is aware of mental illness and stigma is reduced	Improved mental health literacy and decrease in stigma Community members are aware of local availability of treatment Decreased reported stigma by people with priority disorders	Community survey Cohort

q. Environmental, policy, social and political context of the district is monitored for modification of implementation	Changes in environmental, policy, social and political contexts are monitored throughout implementation	Case study: district profile

#### Outcomes pathway

The PRIME ToC identifies political buy-in as the first step in the implementation of the PRIME MHCPs. A guiding principle of the PRIME MHCPs is full integration into the existing district health system with services provided by existing human resources therefore the approval of the MHCP by district health management is necessary for implementation. Next, the ToC identifies the importance of the availability of programme resources necessary for implementation. This includes the availability of medications through a functioning supply chain, human resources to coordinate, train, supervise and deliver services as well as a functioning health information system to measure service delivery.

The need for capacity building is identified by the PRIME ToC for service providers at three levels: specialist, primary healthcare and community. Primary- and community-level service providers need to be competent in the identification or diagnosis of priority mental disorders and should be able to treat or refer where appropriate as well as promoting stigma reduction and increasing awareness of mental illness. The PRIME ToC makes explicit that specialist service providers should be aware that their role in mental health services integrated in primary care includes supervision and training in addition to direct service delivery.

Identification of people with mental disorders is a key outcome in the PRIME ToC and occurs at two levels of the health system: at the community level and at the facility level. This is followed by treatment, care and rehabilitation. For this to occur, the ToC specifies that medications, psychosocial interventions and components of community-based rehabilitation need to be available at the facility and in the community. These interventions need to be acceptable, affordable, accessible, cost-effective and people with priority disorders need to be willing to receive them. They should be delivered for the required duration and individuals should be referred as necessary to other services. To do this, an effective interface between community, facility and specialist services is necessary. To ensure that identification, treatment and care of people with priority disorders occurs at the community and facility levels, adequate ongoing monitoring and evaluation, quality control and supervision is necessary.

If all of the outcomes described in the ToC are achieved, people living with priority disorders treated by the programme and their families or carers should have improved health, social and economic outcomes. If services were scaled up throughout the district resulting in an increase in treatment coverage of the PRIME MHCPs, the desired impact of improving outcomes for people with priority disorders in the whole district should ultimately be achieved, although this impact is beyond the ceiling of accountability for the PRIME programme.

#### Interventions

The content of the interventions required to move from one outcome to the next varies between the different district-level ToCs. This reflects the reality of the MHCPs, the content of which varies between district sites because of differences in the acceptability and feasibility of the interventions that make up the MHCPs. For example, the same outcome, ‘People with mental disorders are identified and/or diagnosed in facilities’ is achieved through different interventions in different districts. In Ethiopia, Uganda, India and Nepal depression is being detected through an adapted version of the WHO mhGAP,^24^ whereas in South Africa mhGAP has been included in national integrated guidelines for chronic care at primary-healthcare-level, called Primary Care 101 (PC101).^26^ Similarly, the outcome ‘People with priority disorders receive treatment as intended for the required duration and are adequately referred if necessary’ is achieved through different interventions in each district. In South Africa this is being provided by psychosocial rehabilitation groups,^26^ whereas in Ethiopia this will be provided by individual community-based rehabilitation.^22^ In Uganda, Ethiopia and India, people with priority disorders will be referred to existing community organisations or non-governmental organisations providing rehabilitation.^22,23,25^ In Nepal, community counsellors will be delivering individual- and family-based psychological treatments.^24^ The content of these interventions, the supporting evidence base and human and other resources required to implement the MHCPS are detailed in other papers in this supplement^22–26^ and compared across all five PRIME districts by Hanlon *et al.*^7^

#### Assumptions

The cross-country ToC makes explicit several assumptions about what needs to be in place for the outcome pathway to be achieved. These include political buy-in that results in adequate funding, committed leadership at various levels and engagement of staff at all levels in the programme despite the lack of financial incentives available. For all levels of service providers, the ToC specifies that there needs to be relative stability within the human resources so that trained staff are retained or new staff are trained in order for the ToC to achieve its stated outcomes.

#### Indicators

The short-, medium- and long-term outcomes of the ToC map roughly divide into inputs (political buy-in, programme resources), processes (capacity building), outputs (identification, treatment, rehabilitation and care) and outcomes of the PRIME MHCPs. Each ToC outcome is operationalised and measured by an indicator. For example, the outcome ‘Essential medications are available in health facilities’ is being measured by the indicator ‘Medications are available at all clinics 95% of time (disaggregated by clinic and type of medication)’. ‘People with mental disorders are identified in the community’ is being measured by ‘Increased number of cases detected and managed by community health workers’. Indicators for the summary ToC are shown in Tables 3 and 4.

Four major study designs were developed to collect data for these indicators and are described in detail in this supplement by De Silva *et al.*^28^ These include:

- Repeat cross-sectional community surveys conducted at baseline and 24 months after MHCP implementation in four of the districts where PRIME will be implemented.
- Repeat facility surveys conducted at baseline, 3–6 months and 24 months after implementation of the PRIME MHCPs in all study districts.
- Treatment cohort for the PRIME priority disorders in all study districts.
- A case study in all study districts including profiles of the community, facility and healthcare organisation, qualitative process evaluation of the MHCPs, evaluations of training quality and fidelity, and costing of the MHPCs.

### Comparison of cross-country ToC and between district-specific ToCs

In general, there is a lot of similarity both between the cross-country ToC and between the district-specific ToCs. Specifically, the temporal dimension of the district ToCs are similar to each other and the cross-country ToC. Although not always explicitly identifying the underlying matrix as the cross-country ToC does, all ToCs identify outcomes related to political buy-in, programme resources, identification of people with priority mental disorders, treatment, care and rehabilitation and the long-term outcome and impact. The vertical axis of the cross-country ToC matrix, i.e. the level of implementation, are also reflected either explicitly or implicitly in all the district ToCs. There are some differences between the district ToCs in relation to the specific outcomes required to reach the impact. These are a result of the discussions at the ToC workshop and other formative work. For example, in India, recommendations from the ToC workshop include the establishment of a dedicated mental health cell to coordinate mental health services at facility level. In Nepal, adolescent depression was identified as a priority area therefore they have added an outcome requiring a functioning psychosocial support programme to be in place in schools. As discussed above, the interventions that comprise the MHCPs differ in each country based on feasibility and acceptability. These differences are also reflected in interventions outlined in the district-specific ToCs.

The majority of assumptions in the district-specific ToCs are similar between countries and related to issues of political buy-in, budget, the willingness and capacity of staff to participate in training and service delivery and the willingness of other organisations to provide services. However, there are some differences according to country. For example, in South Africa, where there is a relatively good supply of psychotropic medication, there was no need to intervene. Therefore, rather than including it as an outcome in the South African ToC it is listed as an assumption. The indicators used to measure success differ between countries based on the country-specific outcomes, the availability of routine data, feasibility of data collection and whether these indicators are being measured as part of the cross-country evaluation design.

## Discussion

### Main findings

The ToC approach led to the development of an underlying programme theory, highlighting the outcomes required for the integration of mental health into primary healthcare at district level. The PRIME ToC map provides a visual summary of the programme, making explicit the hypothesised causal pathways through which the components of the MHCP interact to achieve the intended long-term outcome of improved clinical, social and economic outcomes for people with priority mental disorders.

The underlying programme theory is similar across all the country ToCs and can be summarised by the cross-country ToC. We hypothesise various reasons for this. First, the development of the cross-country and district ToCs were interlinked. Facilitators from countries were involved in the conceptualisation of the PRIME goals and guiding principles and the development of the cross-country ToC. Following the development of the district ToCs the cross-country ToC was revised to ensure that that the cross-country ToC reflected the main causal pathways outlined in the district ToCs. Second, there are similarities between PRIME implementation districts such as the low coverage of evidence-based mental health services, lack of integrated mental health services at primary care level and their associated support structures, a paucity of mental health specialists and low levels of financial support for mental health.^3,20^

### Findings from other studies

The PRIME ToCs are similar to other ToCs used to plan mental health services in which the authors are involved.^34^ ToC was used to develop a counselling intervention for maternal depression delivered by community health workers in Pakistan by the South Asian Hub for Advocacy, Research and Education on mental health (SHARE) and to develop a community-based rehabilitation intervention for a randomised controlled trial, Rehabilitation Intervention for People with Schizophrenia in Ethiopia (RISE).^34^ In both SHARE and RISE the temporal dimension of the ToC matrices are similar to PRIME, including outcomes related to programme resources, capacity building, identification, treatment, care and rehabilitation, long-term outcome and impact. As both SHARE and RISE are primarily focused at one level of the health system they do not make explicit the vertical dimension of their ToC specifying the levels of the health system. However, they do include referral to other levels of the health system within the causal pathway of their ToC. The actual outcomes along the causal pathway differ between SHARE, RISE and PRIME with SHARE and RISE providing more detailed outcomes given the more narrow focus of their programme. The similarities of these three ToCs indicate that the PRIME ToCs may capture the programme theory underpinning the provision of mental health services integrated into the health system. Therefore the cross-country ToC could be used as a heuristic device to aid the development and scaling up of mental health services in similar settings.

### Importance of workshops

As described in detail elsewhere,^27^ the process of developing PRIME-country ToCs through participatory workshops contributed to the development of contextually relevant PRIME MHCPs with the buy-in of a broad range of stakeholders (Table 1). The stepwise approach to ToC development allowed stakeholders to discuss in detail the hypothesised outcomes required along the causal pathway and ensured that the initial focus of the workshops remained on the outcomes that needed to be achieved. Although the underlying programme theory identifying the required outcomes was similar across all sites, the substance of the interventions that formed the basis of the MHCPs and details of implementation such as the cadre of human resources delivering the intervention, type and location of the intervention varied between countries. During this process, the assumptions of various stakeholders and potential challenges in implementation were explored. Brainstorming around solutions to these challenges with various stakeholders in the district allowed local solutions to be recommended. For example, in Nepal, where the supply of psychotropic medications is erratic, policy makers suggested alternate solutions to ensure a regular medication supply. The presence of a wide range of stakeholders such as district management, planners, policy makers, service providers and researchers allowed stakeholders to work together to plan the impact they want to achieve and to ensure ownership of the MHCPs.^35^ Feedback from the facilitators of the workshops indicated that stakeholders were engaged in the ToC process,^25^ however, it is too early to establish whether participation in the workshops led to sustained engagement in the project and support during implementation.

### Use of ToC in evaluation

The cross-country ToC also provided a useful framework to develop the evaluation design for the MHCPs. Once the indicators had been identified for all the outcomes in the PRIME ToC they were operationalised into cross-country study designs.^28^ An advantage of the ToC is the focus on measuring indicators for each outcome on the ToC pathway resulting in a clear evaluation of inputs, processes and outcomes across the whole causal pathway of the intervention. This helps to unpack the black box of a complex intervention by distinguishing intervention ineffectiveness from implementation failure and assesses the relative contributions of specific components of the MHCPs to the overall outcome.^34^ As the same outcomes are being measured across all sites it allows us to compare the effectiveness of the components of the MHCPs across sites. This is particularly important for the evaluation of complex, multisite interventions such as PRIME.

Another important aspect of the evaluation of complex interventions is the influence of context on the implementation and outcomes.^36^ The PRIME ToC makes explicit the need to measure the influence of context on achieving the pathway to having an impact. This is important because the contextual conditions in each PRIME country vary significantly and are influenced by other social, political and health system changes. The Dr Kenneth Kaunda district in South Africa, for example, is well resourced compared with other countries^20^ and is a pilot site of other government-led initiatives such as the introduction of primary healthcare re-engineering and an integrated chronic disease management model of care. In Sehore, India, there is the concurrent introduction of the district mental health programme whereas in Sodo, Ethiopia and Chitwan, Nepal, there are currently no major initiatives with regard to mental health. Consequently, careful documentation and analysis of context in the case study will be essential to interpret the results of the PRIME evaluation of the MHCPs.

### Limitations

The PRIME ToC can be used as a heuristic device that is adapted and refined to implement and scale up MHCPs in similar settings. This may increase the efficiency of the ToC process^37^ but may compromise the stakeholder buy-in and bottom–up development of the ToC that we found in our ToC workshops.^27^ We therefore recommend that ToC workshops are still held as part of the planning process to ensure ownership of a larger group of stakeholders that may increase the chance of successful implementation.^35^

Although the PRIME experience has shown that the ToC process may be useful for the development of MHCPs and planning their evaluation, the PRIME ToC does provide a simplistic framework of a complex health intervention. PRIME is likely to possess the characteristics of complex systems such as recursive causality, tipping points and emergent outcomes, which have not been expressed explicitly in the PRIME ToC.^38^ We have taken this into account in the analysis of context, as mentioned in the ToC. However, the cross-country ToC still focuses on health services and may inadvertently miss causal pathways leading to unintended consequences of the intervention, for example, the effects of socioeconomic changes on individuals that are not captured by the PRIME evaluation.^38^ Areas for further research include refining the methods for using ToC to design and evaluate mental health programmes, adaptation of the ToC method for the scale up of mental health services and testing the use of ToC as a framework for combining process and outcome evaluations.^29^

In conclusion, using ToC can assist in planning mental health services. In a multicountry programme evaluating the integration of mental health into primary healthcare (PRIME) we developed a cross-country ToC and district-specific ToCs with diverse stakeholders. The district-specific ToCs formed the basis of the MHCPs in each district and the cross-country ToC provided a framework to identify indicators for key outcomes along the causal pathway of the MHCPs. This in turn informed the development of the PRIME evaluation design. The cross-country ToC may be a useful heuristic device that can be used and adapted by other programmes when planning the integration of mental health into primary care in low-resource settings.
